# MLACP: machine-learning-based prediction of anticancer peptides

**DOI:** 10.18632/oncotarget.20365

**Published:** 2017-08-19

**Authors:** Balachandran Manavalan, Shaherin Basith, Tae Hwan Shin, Sun Choi, Myeong Ok Kim, Gwang Lee

**Affiliations:** ^1^ Department of Physiology, Ajou University School of Medicine, Suwon, Republic of Korea; ^2^ College of Pharmacy, Graduate School of Pharmaceutical Sciences, Ewha Womans University, Seoul, Republic of Korea; ^3^ Institute of Molecular Science and Technology, Ajou University, Suwon, Republic of Korea; ^4^ Division of Life Science and Applied Life Science (BK21 Plus), College of Natural Sciences, Gyeongsang National University, Jinju, Republic of Korea

**Keywords:** anticancer peptides, hybrid model, machine-learning parameters, random forest, support vector machine

## Abstract

Cancer is the second leading cause of death globally, and use of therapeutic peptides to target and kill cancer cells has received considerable attention in recent years. Identification of anticancer peptides (ACPs) through wet-lab experimentation is expensive and often time consuming; therefore, development of an efficient computational method is essential to identify potential ACP candidates prior to *in vitro* experimentation. In this study, we developed support vector machine- and random forest-based machine-learning methods for the prediction of ACPs using the features calculated from the amino acid sequence, including amino acid composition, dipeptide composition, atomic composition, and physicochemical properties. We trained our methods using the Tyagi-B dataset and determined the machine parameters by 10-fold cross-validation. Furthermore, we evaluated the performance of our methods on two benchmarking datasets, with our results showing that the random forest-based method outperformed the existing methods with an average accuracy and Matthews correlation coefficient value of 88.7% and 0.78, respectively. To assist the scientific community, we also developed a publicly accessible web server at www.thegleelab.org/MLACP.html.

## INTRODUCTION

Cancer is a heterogeneous group of several complex diseases, rather than a single disease, which is characterized by uncontrolled cell growth and the ability to rapidly spread or invade other parts of the body. This inherent complexity and heterogeneous nature of cancer has proven to be a major hurdle for the development of effective anticancer therapies [1]. Conventional methods for cancer treatment, including radiotherapy and chemotherapy, are expensive and often exhibit deleterious side effects on normal cells. Additionally, cancer cells are capable of developing resistance to current anticancer chemotherapeutic drugs [2, 3]. Therefore, it is necessary to continually develop novel anticancer drugs to attenuate cancer cell proliferation. Peptide-based therapy has several advantages over the use of other small molecules due to their high specificity, increased capability for tumor penetration, and minimal toxicity under normal physiological conditions [4].

Anticancer peptides (ACPs) are peptides capable of use as therapeutic agents to treat various cancers. Recent studies showed that ACPs are selective toward cancer cells without affecting normal physiological functions, making them a potentially valuable therapeutic strategy [5, 6]. ACPs contain between 5-30 amino acids and exhibit cationic amphipathic structures capable of interacting with the anionic lipid membrane of cancer cells, thereby enabling selective targeting [7, 8]. In the previous decade, multiple peptide-based therapies against various tumor types have been evaluated and are currently undergoing evaluation in various phases of preclinical and clinical trials [9], confirming the importance of developing novel ACPs for cancer treatment.

Experimental identification and development of novel ACPs represent extremely expensive and often time-consuming processes. Therefore, development of sequence-based computational methods is necessary to allow the rapid identification of potential ACP candidates prior to their synthesis. To this end, computational methods, including AntiCP, iACP, and that described by Hajisharifi *et al* (2014), have been developed for ACP prediction [10–13]. Existing methods separately use properties, such as amino acid composition (AAC), binary profile, dipeptide composition (DPC), and Chou's pseudo-amino acid composition (PseAAC), extracted from the primary sequence as input features to a support vector machine (SVM) for the development of a prediction model. Surprisingly, all of these methods use the same machine-learning (ML) method, with the two methods [that of Hajisharifi *et al* (2014) and iACP] using the same dataset for prediction-model development. These methods produced encouraging results, and iACP and AntiCP remain the only publically available programs for assisting the scientific community [14–16].

Although, the existing methods have specific advantages for ACP prediction, it remains necessary to improve prediction accuracy. In this study, we developed ML-based methods [SVM and random forest (RF); named SVMACP and RFACP, respectively] to predict ACPs (MLACP) using combinations of features calculated from the peptide sequence, including AAC, DPC, atomic composition (ATC), and physicochemical properties (PCP). When tested upon benchmarking datasets, our proposed methods outperformed the existing ones in predicting ACPs. Moreover, we developed a web tool to assist the scientific community working in the field of ACP therapeutics and biomedical research.

## RESULTS

### Dataset construction

**Figure 1:**
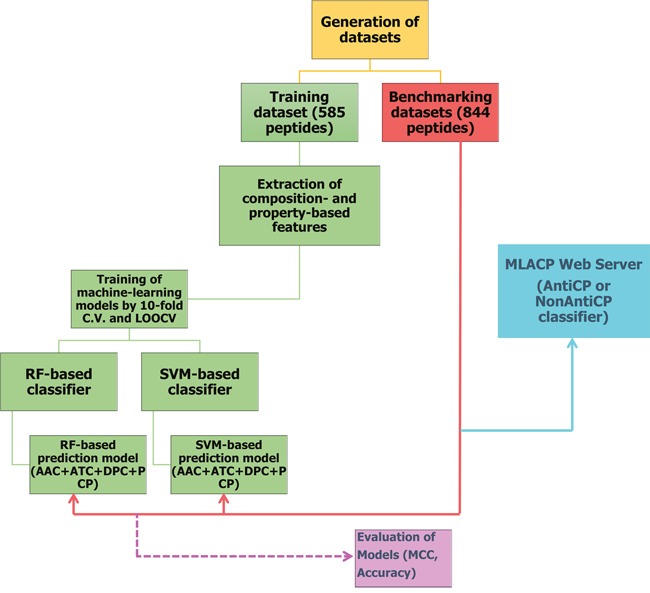
Flowchart showing steps involved in the development of prediction model (MLACP methodology)

**Figure 2:**
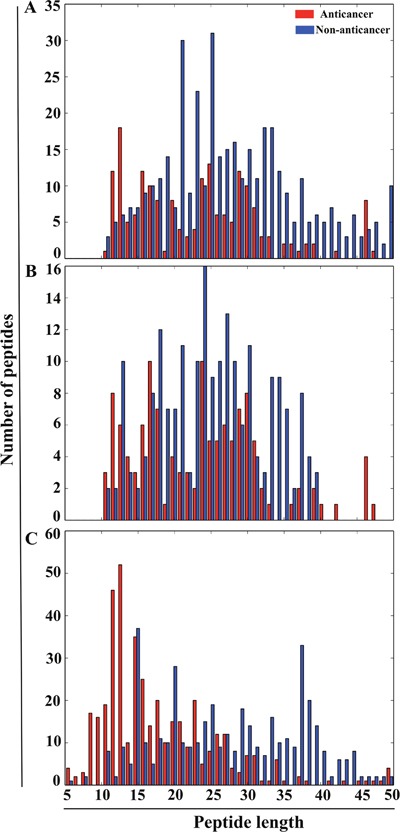
Histogram of the peptide-length distribution of ACPs and non-ACPs X- and Y-axes represent peptide length and number of peptides. **(A)** Tyagi-B dataset. **(B)** HC dataset. **(C)** LEE dataset.

A detailed description of dataset construction is given in the ‘materials and methods’ section. An overview of our methodology is shown in Figure 1. Briefly, we generated three different datasets, namely Tyagi-B dataset, Hajisharifi-Chen (HC), and LEE dataset. The histogram of peptide-length distribution of these datasets is shown in Figure 2. Most of the ACPs contain <35 amino acid residues and non-ACPs have a wider size distribution in Tyagi-B dataset (Figure 2A), which was utilized in the development of a prediction model. HC and LEE datasets were treated as benchmarking datasets. Among these, HC showed similar distribution between ACPs and non-ACPs (Figure 2B), whereas, in LEE dataset, most of the ACPs contained <25 amino acid residues and non-ACPs showed a wider distribution (Figure 2C).

### Compositional analysis

**Figure 3:**
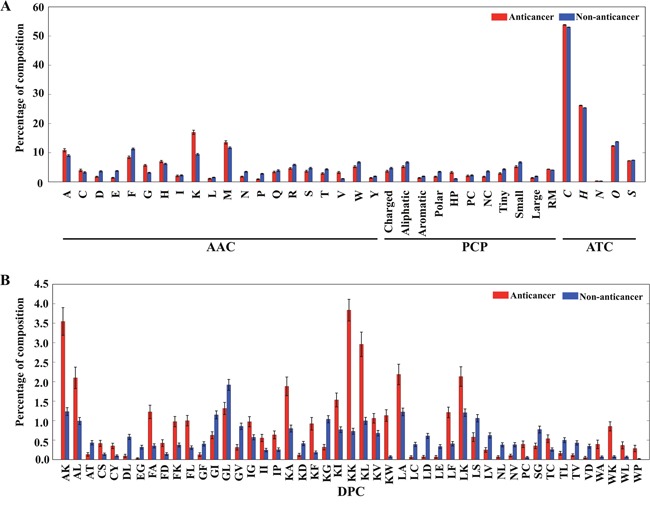
Comparison of AAC, ATC, PCP, and DPC features between ACPs and non-ACPs **(A)** Three different compositions (AAC, PCP, and ATC). For PCPs, HP, PC, NC, and RM represent hydrophobic, positively charged, negatively charged residues and residue mass, respectively. To discriminate element in ATC from AAC, we have shown in italics. Similarly, for PCP to discriminate from DPC. **(B)** For DPC, we showed only dipeptides exhibiting the absolute differences between ACP and non-ACP is greater than 0.25.

To perform compositional analysis of ACPs and non-ACPs, AAC, DPC, PCC, and ATC frequencies were calculated using the Tyagi-B and HC datasets. AAC analysis revealed that certain residues, including A, F, K, L, and W, were dominant in ACPs, whereas D, E, G, N, and Q were dominant in non-ACPs (Welch's *t* test; *p* < 0.01). PCP analysis indicated that only two properties (hydrophobicity and residue mass) were dominant in ACPs, whereas the remaining nine properties were dominant in non-ACPs. ATC analysis revealed that hydrogen and carbon content dominated at a slightly higher level in ACPs as compared with non-ACPs (Figure 3A). Moreover, DPC analyses revealed that 104 out of 400 dipeptides were differentially present in ACPs and non-ACPs (*p* < 0.01). Our analyses also revealed that the 10 most abundant dipeptides in ACPs and non-ACPs were KK, AK, KL, AL, KA, KW, LA, LK, FA, and LF and KG, GL, GV, LD, GI, DL, LS, SG, LV, and TL, respectively (Figure 3B).

Based on these findings, it was evident that the most abundant dipeptides in ACPs consisted primarily of pairs of positively charged-aromatic or –aliphatic amino acids, positively charged-positively charged amino acids, or aliphatic-aromatic amino acids, whereas the most abundant dipeptides in the non-ACPs were pairs of aliphatic–negatively charged amino acids and aliphatic–hydroxyl-group-containing amino acids. As expected, these results agreed with AAC analysis, which showed that positively charged and aromatic amino acids were abundant in ACPs, whereas negatively charged and hydroxyl-group-containing amino acids were the most abundant in non-ACPs.

### Construction of SVMACP and RFACP

**Figure 4:**
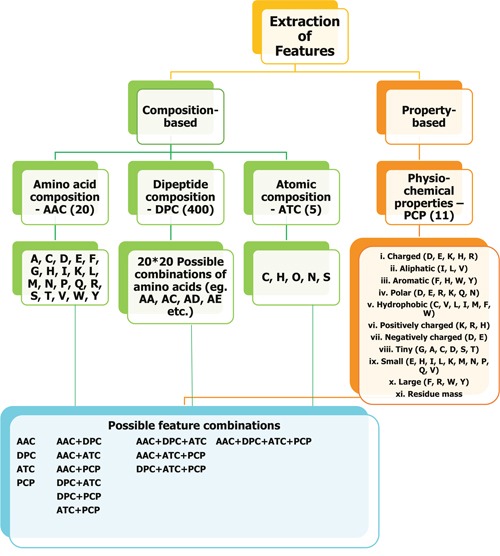
Overview of feature extraction We used both composition-based and property-based information from a given peptide sequence and used as input feature to ML method. AAC, DPC, ATC, and PCP contained 20, 400, 5, and 11 features, respectively.

In this study, we considered two most commonly used ML methods (*i.e*. RF and SVM) to predict ACPs. One of the most important steps in ML method is feature selection. Here, we considered both composition- and property-based features (Figure 4). AAC, DPC, ATC, and PCP contained 20, 400, 5, and 11 features, respectively. First, we developed a prediction model based on an individual composition and subsequently developed hybrid models based on the combination of all possible compositions. For each model, we optimized the ML parameters (SVM: C and γ; RF: *ntree, mtry*, and *nsplit*) by using 10-fold cross-validation on Tyagi-B dataset. During 10-fold cross-validation, the Tyagi-B dataset was randomly divided into 10 parts (with ~10% ACPs and non-ACPs resident in each part), from which nine parts were used for training, and the 10^th^ part was used for testing. This process was repeated until all the parts were used at least once as a test set, and the overall performance on all 10 parts was evaluated. The optimal parameters which gave the highest MCC was selected as the final one. It should be noted that we performed ten independent 10-fold cross-validations to verify the robustness of the ML parameters.

The following subsections describe the development of different models and the criteria used for final-model selection.

### AAC-based models

Previous studies showed that AAC-based ML methods had been developed for the classification of different classes of peptides [14, 16]. During compositional analysis, we found significant differences between ACPs and non-ACPs (Figure 3). Therefore, we utilized these differences to classify peptides as ACPs or non-ACPs using ML models. Table 1 shows that the SVM model produced the best classification, with an accuracy of 0.858 and an MCC of 0.664, while the corresponding values for the RF model were 0.868 and 0.689, respectively.

**Table 1:** Performance of various prediction models on training dataset

Features	MCC		Accuracy		Sensitivity		Specificity	
	SVM	RF	SVM	RF	SVM	RF	SVM	RF
AAC	0.664	0.689	0.858	0.868	0.695	0.706	0.935	0.945
ATC	0.519	0.587	0.802	0.826	0.503	0.658	0.942	0.905
PCP	0.420	0.553	0.759	0.814	0.524	0.599	0.869	0.915
DPC	0.653	0.644	0.853	0.850	0.706	0.599	0.922	0.967
AAC+ATC+PCP+DPC	**0.697**	0.698	0.872	0.872	0.706	0.722	0.95	0.942
AAC+PCP+DCP	0.693	0.661	0.870	0.856	0.706	0.620	0.947	0.967
AAC+PCP+ATC	0.685	0.698	0.867	0.872	0.695	0.727	0.947	0.940
AAC+PCP	0.681	0.681	0.865	0.865	0.695	0.695	0.945	0.945
AAC+ATC	0.664	0.673	0.858	0.862	0.695	0.642	0.935	0.965
AAC+DCP	0.673	0.657	0.862	0.855	0.701	0.61	0.937	0.970
PCP+ATC+DCP	0.661	0.669	0.856	0.86	0.711	0.631	0.925	0.967
PCP+ATC	0.595	0.664	0.831	0.858	0.615	0.685	0.932	0.940
PCP+DCP	0.661	0.661	0.856	0.856	0.701	0.620	0.93	0.967
ATC+DCP	0.657	0.661	0.855	0.856	0.701	0.620	0.927	0.967

The first column represents the features. The second, the third, the fourth and the fifth respectively represent the MCC, accuracy, specificity and sensitivity. Columns 2-5 subdivided into two parts namely SVM- (normal font) and RF-based (underlined) performances. AAC: amino acid composition; ATC: atomic composition; PCP: physiochemical properties; DPC: dipeptide composition. Features that gave the highest MCC is shown in bold.

### DPC-based models

DPC provides additional information regarding the global and local arrangement of residues in a sequence as compared with AAC. DPC-based ML methods have been previously utilized to classify different classes of peptides [17–19]. Therefore, in this study, we developed RF- and SVM-based models using DPCs. The SVM model produced the best classification, with an accuracy of 0.853 and an MCC of 0.653, whereas the corresponding values for the RF-based model were 0.850 and 0.644, respectively. The performance of the DPC-based model was similar to that of the AAC-based model.

### ATC-based models

We calculated a set of ATCs from the given peptides, because these were previously shown to be useful for the prediction of antihypertensive peptides [17–19]. Therefore, in this study, we developed RF- and SVM-based models using ATC. Our results showed that the SVM-based model produced the best classification, with an accuracy of 0.802 and an MCC of 0.519, whereas the corresponding values for the RF-based model were 0.826 and 0.587, respectively. However, the performance of the ATC-based model was slightly worse relative to that of the AAC- and DPC-based models (Table 1).

### PCP-based models

For each dataset, we calculated a set of PCPs for each peptide, because these were previously shown to be useful for the prediction of different classes of proteins [19]. Therefore, in this study, we developed SVM- and RF-based models using PCPs. Results indicated that the SVM-based model produced the best classification, with an accuracy of 0.759 and an MCC of 0.420, whereas the corresponding values for the RF-based model were 0.814 and 0.553, respectively. However, the performance of this model was worse relative to that of each of the other three models (Table 1).

### The hybrid model

**Figure 5:**
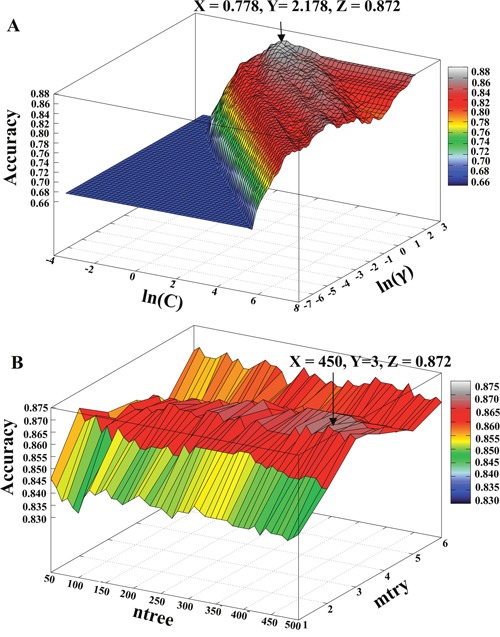
Accuracies obtained from 10-fold cross-validation using various parameters **(A)** The X- and Y-axes represent the SVM parameters *C* and γ on a natural logarithmic scale. The Z-axis represents the accuracy with respect to variations in *C* and γ. **(B)** The X- and Y-axes represent the RF parameters *ntree* and *mtry*. The Z-axis represents the accuracy with respect to variations in the parameters *ntree* and *mtry*. The arrow represents the maximum accuracy.

Although individual composition-based models showed good or acceptable performance, to further improve the collective performance, we combined these features using all possible combinations to construct hybrid models. This approach has been widely applied in different peptide- and protein-composition-based classification methods [20, 21]. Table 1 shows that a hybrid model containing all of the composition- and property-based features produced the best classification among the different SVM-based hybrid models. Figure 5A shows the profile of classification accuracy verses the variations of parameters *C* and *γ* using all composition- and property-based features. The best classification accuracy of 0.872 peaked at (ln(C), ln(*γ*)) = (0.778, 2.178) was selected as the final model (SVMACP). Moreover, Table 1 shows that an RF-based hybrid model containing all of the features and a model containing only three features (excluding DCP) produced the same results. Notably, adding DCP features into the three combined features did not detract from the predictive performance; therefore, we selected the model containing all of the composition- and property-based features as the final prediction model (RFACP). Figure 5B shows the profile of classification accuracy verses variations in the parameters *ntree* and *mtry* using all composition- and property-based features. The best classification accuracy of 0.872 peaked at (*ntree*, *mtry*) = (450, 3) was selected as the final RF-based model.

### Performance of our methods against AntiCP using the HC dataset

We evaluated the performance of our methods (SVMACP and RFACP) against that of the AntiCP (model_1 and model_2) using the HC dataset, with the results shown in Table 2. The methods in the Table 2 are ranked according to the accuracy, which reflects the prediction capability of the method. For comparison, we also included iACP and the methods presented by Hajisharifi *et al* (2014) results, wherein the authors used the same dataset for their prediction model development [22]. Among the methods evaluated using the HC dataset, RFACP ranked at the top, with MCC, accuracy, sensitivity, and specificity values of 0.885, 0.946, 0.889, and 0.981, respectively. Additionally, RFACP performance was significantly better than that of AntiCP models, which exhibited ~8% and ~54% decreases in model_2 and model_1 accuracy, respectively, and SVMACP, which exhibited an ~6% decrease in accuracy. Furthermore, comparison of RFACP relative to iACP and that of Hajisharifi *et al* (2014) showed that RFACP results were slightly better than those of the method presented by Hajisharifi *et al* (2014), which exhibited a decrease in accuracy of ~2%, and similar to iACP results. Table 2 shows that SVMACP ranked second among all of the methods, exceeding the performance of the AntiCP models, which exhibited ~1% and ~48% decreases in accuracy for model_2 and model_1, respectively. When comparing both AntiCP models, it was observed that model_1 predicted almost all of the given peptides as potential ACPs, suggesting that model_2 performance is better in ACP prediction.

**Table 2:** Performance of various methods on the HC dataset

Method	MCC	Accuracy	Sensitivity	Specificity
iACP*	0.897	0.951	0.899	0.985
RFACP	**0.885**	**0.946**	0.889	**0.981**
Hajisharifi et al*.	0.784	0.927	0.897	0.852
SVMACP	0.750	0.882	0.841	0.907
AntiCP (Model_2)	0.719	0.869	0.813	0.902
AntiCP (Model_1)	0.062	0.402	**0.976**	0.049

The first column represents the method name. The second, the third, the fourth, and the fifth respectively represent the MCC, accuracy, sensitivity and specificity. For comparison, we also included iACP and Hajisharifi *et al*. results, which is based on the training dataset results (*). Bold font denotes the best result.

### Performance of our methods and other existing methods using the LEE dataset

We evaluated the performance of our methods (SVMACP and RFACP), and the existing methods including iACP, and AntiCP (model_1 and model_2) on the LEE dataset. Notably, our LEE dataset contained 844 peptides, which was ~3-fold larger than previously used benchmark datasets. Table 3 shows that RFACP was ranked at the top, with MCC, accuracy, sensitivity, and specificity values of 0.674, 0.827, 0.706, and 0.948, respectively. Additionally, the performance of RFACP was slightly better than that of SVMACP, which showed a ~1% decrease in accuracy, and significantly better than AntiCP models, which exhibited ~7.5% and ~30% decreases in accuracy for model_2 and model_1, respectively, and iACP, which exhibited ~12% decreases in accuracy. SVMACP ranked second in performance, which was significantly better than AntiCP models, which exhibited ~6% and 28.7% decreases in accuracy for model_2 and model_1, respectively, and iACP, which exhibited 11% decreases in accuracy. AntiCP model_2 and iACP occupied the third and fourth positions, respectively, with AntiCP model_1 exhibiting the worst performance. This evaluation clearly showed that RFACP and SVMACP exceeded the performance of the existing methods. Interestingly, although SVMACP and RFACP produced the same results (MCC: 0.697 and 0.872, respectively) on the training dataset, RFACP performance was slightly better on the benchmarking datasets (~6% better on the HC dataset and ~1% better on the LEE dataset) relative to that of SVMACP. This result showed that the RF-based method was more effective than the SVM for ACP prediction. A previous study reported successful application of RF for many biomedical classification problems [14, 15, 23]. Moreover, a detailed comparison of our methods and the existing methods in terms of methodology is provided in Table 4, showing that our methodology exceeded current methods while using a slightly larger training dataset, different ML methods, additional features, and larger benchmarking datasets.

**Table 3:** Performance of various methods on the LEE dataset

Method	MCC	Accuracy	Sensitivity	Specificity
RFACP	**0.674**	**0.827**	0.706	**0.948**
SVMACP	0.630	0.814	0.775	0.853
AntiCP (Model_2)	0.505	0.752	0.744	0.761
iACP	0.412	0.706	0.697	0.716
AntiCP (Model_1)	0.096	0.527	**0.938**	0.116

The first column represents the method name. The second, the third, the fourth, and the fifth respectively represent the MCC, accuracy, sensitivity and specificity. Bold font denotes the best result.

**Table 4:** A comparison of anticancer peptide prediction methods

Method	Choice of ML method	Cross-validation	Training dataset size	Benchmarking dataset size	Features
AntiCP	SVM	10-fold cross-validation (10-fold CV)	450	200	AAC, DPC, and binary profile
iACP	SVM	Leave-one-out cross-validation (LOOCV)	344	300	one-gap DPC
Hajisharifi *et al*.	SVM	LOOCV	344	22	Chou's PseAAC
MLACP	SVM and RF	10-fold CV	585	332 and 603	AAC, DPC, ATC, and PCP

The first column represents the method name. The second column represents the choice of ML methods used for their method development. The third column represents the cross-validation procedure used for the optimization of ML parameters. The fourth and fifth column respectively represent the size of the training dataset and benchmarking dataset. The final column represents the total number of compositional features considered by each method. AAC: amino acid composition; ATC: atomic composition; PCP: physiochemical properties; DPC: dipeptide composition.

### The MLACP online prediction server

As mentioned in a series of publications [20, 24–30], a prediction method along with its web server would be practically useful to the experimentalists [31–37]. To this end, an online prediction server called MLACP was developed to allow ACP prediction using the methods presented here. The prediction server is freely accessible at the following link: www.thegleelab.org/MLACP.html. Users can paste or upload query peptide sequences in the FASTA format, and after submitting peptide sequences, retrieve results in a separate interface. To enable the reproducibility of our findings, all datasets used in this study can be downloaded from the MLACP web server.

## DISCUSSION

Anticancer peptides exhibit a broad spectrum of activity, including the ability to kill cancer cells, destroy primary tumors, prevent metastasis, and perform these actions at adequate concentrations without damaging normal cells or vital organs [38]. To identify highly efficient ACPs, an experimentalist should screen a peptide from the existing peptide libraries or scan the entire protein in overlapping-window patterns associated with areas of peptide chains, and test each segment for its potential anticancer activity, which seems laborious and time-consuming. Therefore, the development of sequence-based computational methods capable of determining ACP candidates will be helpful to researchers, who are keen to rapidly screen ACPs prior to its synthesis, thereby accelerating ACP-based research. Here, we developed two MLACP methods, RFACP and SVMACP.

AAC, DPC, ATC, and PCP analyses revealed that ACPs most often consist of positively charged, aromatic, and hydrophobic residues. Previous studies showed that peptide hydrophobicity plays an important role in membrane permeabilization and/or anticancer activity [9, 39]. Furthermore, we observed a significant difference in residue preference between ACPs and non-ACPs, which prompted us to use these as input features to ML methods to encourage improved classification. The major advantage of ML methods is their capability to consider multiple features simultaneously, often capturing hidden relationships [40–46].

In this study, we employed two different ML algorithms, SVM and RF, for ACP prediction, whereas existing methods use only SVM [14, 16]. This is the first application of an RF-based method in ACP prediction, with systematic approaches employed to select between SVMACP- and RFACP-based prediction models. Notably, MLACP represents the only method utilizing a combination of all composition- and property-based features as inputs; however, other existing methods [AntiCP, iACP, and that of Hajishari *et al* (2014)] utilize only one of the following properties, AAC, DPC, binary profile, or PseAAC, separately as an input feature to SVM in order to develop their prediction models [14–16]. Although, AAC and DPC features were used in earlier studies, this is the first study describing the use of PCP and ATC features for ACP prediction. To show the effect of including PCP and ATC in MLACP (*i.e.* RFACP and SVMACP), we evaluated a prediction model (which contains only AAC and DCP as input features) on LEE datasets. Supplementary Information 1 shows that improvement of both ML-based methods is found by adding PCP and ATC into MLACP.

We used two benchmarking datasets (HC and LEE) to evaluate the performance of our methods along with the existing methods. Using the HC dataset, RFACP and SVMACP, respectively, ranked as the first and second most effective predictors, with significantly better performances than the existing AntiCP methods (model_2 and model_1). Interestingly, RFACP accuracy was better than that of the method described by Hajisharifi *et al* (2014) using the same training set. Recently, Chen *et al* (2016) evaluated their method along with the AntiCP method using a smaller benchmarking dataset (300 peptides). Indeed, this was the first instance where ACP-prediction methods were evaluated using standard benchmarking dataset. However, the LEE dataset constructed in this study was almost 3-fold larger than previously reported benchmarking datasets. Such a large-sized benchmarking dataset is sufficient to evaluate the performance of various methods, with our benchmarking results showing that RFACP significantly outperformed existing methods (AntiCP and iACP) both in terms of accuracy and MCC. SVMACP ranked as the second most effective ACP predictor, with performance still significantly better than those of the other existing methods. The improved performance of our methods is primarily due to the larger size of training dataset, rigorous optimization procedures to select ML parameters, inclusion of new features, the combination of various properties, and the choice of ML method. However, a limitation of this method is that the prediction might not be accurate for longer peptides (length > 50 amino acids) due to their exclusion from the training dataset. Although, our current method is focused on the sequence-based prediction, further studies focused on structure-based membrane-peptide interaction is needed

Consensus algorithms combine output from different predictors popular tools used in various fields of bioinformatics; however, these methods remain in the early stages of development for use in ACP prediction. To generate higher confidence in ACP prediction, we have presented the option of considering consensus results from RFACP and SVMACP methods. Similar approaches were recently implemented *via* generation of consensus results to predict ACPs from *Achatina fulica* mucus for further experimentation [14–16].

The comparatively low cost and minimal time required for the *in silico* identification of ACPs when compared to the tedious and expensive experimental procedures make these computational tools more attractive among the scientific community. In this study, we developed a novel method to predict ACPs from the sequence information and our results showed that the prediction accuracy is significantly higher than the existing methods. Our developed MLACP tool is freely available for research use as a web server. We hope that our method will be useful to both experimentalists and computational biologists.

## MATERIALS AND METHODS

As demonstrated by a series of recent publications [24, 47–51] in compliance with Chou's 5-step rule [52], to establish a really useful sequence-based statistical predictor for a biological system, we should follow the following five guidelines: (a) construct or select a valid dataset to train and test the predictor; (b) formulate the biological sequence samples with an effective mathematical expression that can truly reflect their intrinsic correlation with the target to be predicted; (c) introduce or develop a powerful algorithm (or engine) to operate the prediction; (d) properly perform cross-validation tests to objectively evaluate the anticipated accuracy of the predictor; (e) establish a user-friendly web-server for the predictor that is accessible to the public. Below, we are to describe how to deal with these steps one-by- one.

### Dataset collection

### Training dataset

We utilized the Balanced 1 (B1) and Balanced 2 (B2) datasets described previously [53] to generate a new dataset called the Tyagi-B dataset. In total, we obtained 450 ACPs (225 each from B1 and B2) and 450 non-ACPs (225 each from B1 and B2) by combining both the B1 and B2 datasets. Additionally, we applied the following screening procedures on B1 and B2 datasets: 1) peptides that contained non-natural amino acid residues, 2) peptides with length >50 amino acid residues, and 3) redundant and/or similar peptides defined by the CD-HIT program ( http://www.bioinformatics.org/cd-hit/) by applying a 90% sequence-identity cut-off. It should be noted that similar peptides were removed only from the training dataset and not from the benchmarking dataset. To avoid overfitting in the prediction model, we excluded redundant or similar peptides. Since very few peptides have length greater than 50 amino acid residues, we also excluded these peptides to avoid outlier in the prediction model. After the screening procedure, we obtained 187 ACPs and 398 non-ACPs (Tyagi-B dataset) for use in developing the prediction model.

### Benchmarking datasets

To compare our methods with existing methods, we generated two datasets: 1) one based on the dataset reported from previous studies and 2) another based on our own search against the existing databases. We named the first and second datasets as Hajisharifi-Chen (HC) and LEE datasets, respectively. It should be noted that Hajisharifi *et al* (2014) and Chen *et al* (2016) developed their prediction models using the same dataset, which contained 138 ACPs and 206 non-ACPs. After applying the screening procedure described in the previous section, we obtained 126 ACPs and 205 non-ACPs (HC dataset).

Construction of the LEE dataset proceeded as follows. We applied the screening procedure described in the previous section to an independent dataset (ACPs and non-ACPs: 150 peptides each) reported by Chen *et al* (2016), obtaining 140 ACPs and 94 non-ACPs. Furthermore, we extracted 229 and 53 experimentally validated ACPs from CancerPPDB (http://crdd.osdd.net/raghava/cancerppd/) and APD3 (http://aps.unmc.edu/AP/database/antiC.php), respectively [16]. Because few experimentally determined non-ACPs are present in the LEE dataset, we obtained 98 non-ACPs from the Tyagi independent datasets and generated 234 random peptides from Swiss-Prot (http://web.expasy.org/docs/swiss-prot_guideline.html), with these representing a set of non-ACPs for the LEE dataset. This strategy for creating a negative-control dataset was implemented in previous studies [54, 55]. In total, we generated 844 peptides (422 ACPs and 422 non-ACPs; LEE dataset). We note here that the peptides in the LEE dataset are unique (*i.e*., they are present neither in our training dataset nor the prediction models used by previous methods).

### Feature generation

The aim of this experiment was to train either an SVM or RF model to accurately map input features extracted from a peptide primary sequence to predict its class (*i.e*., ACP or non-ACP), which is considered a classification problem. The most crucial part of this task is extraction of a set of relevant features. All possible features used in this study are shown in Figure 4, and the definition of each composition-based feature is provided below.

### AAC

AAC is defined as the fraction of each amino acid present in a given peptide sequence. AAC can be calculated by using the following equation:

$$\text{AAC}\left(i\right)=\;\frac{\text{Frequency of amino acid}\;(i)}{\text{Length of the peptide}},$$ (1)

where *i* can be any natural amino acid. The AAC has a fixed length of 20 features.

### Atomic composition (ATC)

Recently, Kumar *et al* (2015) reported the number and types of atoms present in naturally occurring amino acids. In this study, we utilized those data and calculated the frequency of each atom (C, H, N, O, and S) present in the given peptide sequence. The ATC has a fixed length of five features.

### DPC

DPC represents the total number of dipeptides normalized by all the possible combinations of dipeptides present in the given peptide sequence. DPC has a fixed length of 400 (20 × 20) features which can be calculated using the following equation:

$$\text{DPC }\left(j\right)=\;\frac{\text{Total number of Dipeptide}\;(j)}{\text{Total number of all possible dipeptides}},$$ (2)

where DPC(*j*) is one of 400 possible dipeptides.

### PCP

PCP represents the physicochemical class of residues present in a given peptide sequence. We calculated the percentage composition of polar (D, E, R, K, Q, N), hydrophobic (C, V, L, I, M, F, W), charged (D, E, K, H, R), aliphatic (I, L, V), aromatic (F, H, W, Y), positively charged (H, K, R), negatively charged (D, E), tiny (A, C, D, G, S, T), small (E, H, I, L, K, M, N, P, Q, V), and/or large (F, R, W, Y) amino acid residues, as well as peptide mass [14, 16, 17, 56], and used these eleven properties as an input feature.

To the best of our knowledge, this is the first study where all four properties have been considered in ACP prediction. Notably, PCC and ATC have never been considered prior to this, whereas DPC and AAC have been utilized in existing ML-based methods for ACP prediction [57, 58].

### Methodology

We employed RF- and SVM-based ML methods to develop a prediction model using the Tyagi-B dataset. The description of the two ML methods is provided below.

### RF

RF is an ensemble technique utilizing hundreds or thousands of independent decision trees to perform classification and regression [43, 59, 60] and that has been used for numerous biological applications. A detailed description of the RF algorithm has been reported elsewhere [61]. The three most influential parameters of this algorithm, including the number of trees (*ntree*), number of variables randomly chosen at each node split (*mtry*), and minimum number of samples required to split an internal node (*nsplit*), require optimization. We optimized these parameters using a grid search within the following ranges: *ntree* from 10 to 500, with a step size of 10; *m* from 1 to 7, with a step size of 1; and *nsplit* from 2 to 10, with a step size of 1.

### SVM

The SVM is a well-known supervised-ML technique used for developing both classification and regression models, and a detailed description of an SVM has been reported elsewhere [14–16, 23, 62, 63]. In this study, we experimented with several common kernels, including a linear, a Gaussian radial-basis function (RBF), and a polynomial. Among these, RBF worked best for our purposes. A RBF-SVM requires optimization of two critical parameters: γ, which controls how peaked Gaussians are centered on the support vectors; and *C*, which controls the trade-off between training error and margin size [45, 46, 63]. These two parameters were optimized using a grid search within the following ranges: *C* from 2^−15^ to 2^10^ and γ from 2^−10^ to 2^10^ in log_2_ scale.

In this study, we used SVM and RF as implemented in the scikit-learn package [64–66].

### Cross-validation

In statistical prediction, the following three cross-validation methods are often used to examine a predictor for its effectiveness in practical application: independent dataset test, subsampling test, and jackknife test. However, of the three test methods, the jackknife test is deemed the least arbitrary that can always yield a unique result for a given dataset as elaborated in [21] and demonstrated by Eqs.28-30 in [52]. Accordingly, the jackknife test has been widely recognized and increasingly used by investigators to examine the quality of various predictors [51, 67–69]. However, to reduce the computational time, we adopted the 10-fold cross-validation in this study was done by many investigators [16, 63].

### Evaluation metrics

To measure prediction quality, we used the following four metrics: sensitivity, specificity, accuracy, and the Matthews correlation coefficient (MCC). Since, the conventional formulae of these metrics lacking intuitiveness and not easy-to-understand for most biologist, particularly MCC. Chen et al [25, 70] derived a new set of equations for the above-mentioned metrics based on Chou's symbols used in studying protein signal peptide cleavage site [71]. The new formulae for these metrics are given in equation (3).

$$\{\begin{array}{c} \text{Sensitivity}=\;\left(1-\;\frac{N_{-}^{+}}{N^{+}}\right)\\ \text{Specificity}=\;\left(1-\;\frac{N_{+}^{-}}{N^{-}}\right)\\ \text{Accuracy}=\left(1-\;\frac{N_{-}^{+}+\;N_{+}^{-}}{N^{+}+\;N^{-}}\right)\\ \text{MCC}=\frac{1-\left(\frac{N_{-}^{+}}{N^{+}}+\;\frac{N_{+}^{-}}{N^{-}}\right)}{\sqrt{\left(1+\;\frac{N_{+}^{-}-N_{-}^{+}\;}{N^{+}}\right)\left(1+\;\frac{N_{-}^{+}-N_{+}^{-}\;}{N^{-}}\right)}} \end{array}$$ (3)

where *N*^+^ represents the total number of ACPs investigated, N−+ represents the number of ACPs incorrectly predicted as non-ACPs, N− represents the total number of non-ACPs investigated and N+− represents the number of non-ACPs incorrectly predicted as ACPs. The formulae given in eq (3) is more intuitive and easy-to-understand, particularly for the meaning of MCC, as concurred by a series of studies published recently [25, 29, 48, 50, 72–74]. The set of metrics is valid only for the single-label systems. For the multi-label systems, whose existence has become more frequent in system biology [75] and system medicine [20, 47, 76], a completely different set of metrics is needed as defined in [77].

### Development of a prediction server

An online prediction server was also developed using hypertext markup language and Java script, with a Python script executing in the backend upon submission of peptide sequences in the FASTA format. Users can submit single or multiple sequences containing only standard amino acid residues in FASTA format, after which the MLACP web server outputs the results of RFACP and SVMACP for a given peptide sequence.

### Statistical analysis

The differences in AAC, ATC, PCP, and DPC between ACPs and non-ACPs were analyzed using Welch's *t* test. The data are presented as mean ± standard error (SE). Statistical differences were considered significant at *p* < 0.01, indicates that the difference is statistically meaningful. All statistical analysis was performed using our own script.

## SUPPLEMENTARY MATERIALS FIGURES AND TABLES



## Abbreviations

AAC: Amino acid composition
ACP: Anticancer peptide
ATC: Atomic composition
DPC: Dipeptide composition
HC: Hajisharifi-Chen
MCC: Matthews correlation coefficient
ML: Machine-learning
MLACP: Machine-learning-based prediction of anticancer peptides
PCP: Physico-chemical properties
PseAAC: Pseudo amino acid composition
RF: Random forest
RFACP: Random forest based anticancer peptide prediction
SVM: Support vector machine
SVMACP: Support vector machine based anticancer peptide prediction
