# Supplementary material for: MLACP: machine-learning-based prediction of anticancer peptides
Source: Oncotarget. 2017 Aug 19;8(44):77121–36. doi: 10.18632/oncotarget.20365 (PMC5652333; doi:10.18632/oncotarget.20365)
Supplement: Supplementary file 1 [file oncotarget-08-77121-s001.pdf]

MLACP: machine-learning-based prediction of anticancer peptides

SUPPLEMENTARY MATERIALS

Supplementary Information 1: Performance of MLACP with and without ATC and PCP features

Method	MCC	Accuracy	Sensitivity	Specificity
RFACP	0.674	0.827	0.706	0.948
RFACP without ATC and PCP	0.570	0.767	0.587	0.945
SVMACP	0.630	0.814	0.775	0.853
SVMACP without ATC and PCP	0.588	0.793	0.736	0.849

The first column represents the method name. The second, the third, the fourth, and the fifth respectively represent the MCC, accuracy, sensitivity and specificity.
